# Impact of a Short-Term Domestic Service-Learning Program on Medical Student Education

**DOI:** 10.5334/aogh.2465

**Published:** 2019-06-26

**Authors:** Brian A. Chang, Elizabeth Karin, Zachary A. Davidson, Jonathan Ripp, Rainier P. Soriano

**Affiliations:** 1 Department of Medical Education, The Icahn School of Medicine at Mount Sinai, New York City, NY, US; 2 Department of Medicine, The Icahn School of Medicine at Mount Sinai, New York City, NY, US; 3 Department of Geriatrics and Palliative Medicine, The Icahn School of Medicine at Mount Sinai, New York City, NY, US

## Abstract

**Purpose:**

The number of global health opportunities offered to medical students has increased over the past 20 years. Recognizing the growing prevalence of these experiences, a number of studies have shown that these types of exposures have a significant impact on medical students’ education. However, there is a paucity of literature on the educational impacts of short-term domestic service-learning trips, which can be more accessible due to fewer logistical and financial barriers. This mixed-methods qualitative/quantitative study aims to understand the impact of a domestic one-week service learning program on medical students’ educational development and career choices.

**Methods:**

The authors conducted a qualitative analysis of journal entries written by a cohort of students during a domestic weeklong service trip. They also administered a survey to all students who had participated in the program between 2009–2016.

**Results:**

In 88.6% (n = 31) of the journal entries, students reported learning about border town life, Native American health, and rural medical practice. In 42.8% (n = 15) of entries, participants described experiences they felt would impact their future medical career decisions. The students’ reflections also revealed implicit benefits such as becoming aware of privilege within society (n = 14, 40.0%). The majority of survey respondents reported that the trip improved their medical education and influenced the field and location of their future/current practice.

**Conclusion:**

This study suggests that domestic short-term service-learning trips impact medical students’ immediate educational development and may influence their future career plans. Further investigation into the local community’s perceptions of this service-learning trip will provide greater understanding of the impact on all involved.

## Introduction

The proportion of US medical schools providing students with opportunities for global health training has increased three-fold in the last two decades, from 22.0% in 1991 to 61.0% in 2008 [1,2]. Recognizing the growing prevalence of these experiences, there have been a number of studies that have investigated their educational impact. These studies have shown that global health experiences enhance students’ clinical knowledge, increase awareness of the social determinants of health, and alter their career trajectories [3,4,5,6,7,8]. Additionally, a study of residents who participated on global health service trips while in medical school reported that these experiences have a prolonged positive impact on their cultural competence, adaptability, and communication skills [9].

While there are many perceived benefits for medical students participating on service-learning trips, the majority relate to global health programs that are longer than a week in duration and are international. Medical students who are interested in global health may not have the ability to travel internationally or participate in service-learning trips longer than one week for a variety of reasons. Thus, participation in these short-term service-learning domestic trips may be their only opportunity to pursue global health interests while meeting their other requirements. Published data on the impact of one-week global health programs are scarce. One study showed that participation in these short-term service-learning trips provides implicit insights and lessons regarding ethical and societal issues within global health practices and may stimulate the development of critical reflection on current and future professional roles for student participants. However, this study only evaluated the impact of one-week service-learning trips outside the US and its findings may not be generalizable to all one-week service-learning trips, especially those that are domestic [10].

The Nogales trip at the Icahn School of Medicine at Mount Sinai is a unique one-week domestic global health service-learning experience with the central goal of introducing participants to rural, border, immigrant, and Native American health through interactions with various members of the Nogales, Arizona community. The US-Mexico border divides the area of Nogales into a rural town in Arizona, USA and a mid-sized city in Mexico. Nogales is located in close proximity to the Tohono O’odham nation, the second largest Native American reservation in the country. Nogales’ specific history and location make it an ideal destination to accomplish the trip’s goals.

Prior to the trip, students are selected through a competitive process to participate in this service-learning experience. In the weeks prior to the trip, the participants engage in a preparatory curriculum, which includes presentations in immigration/border issues, preventative health screening practices, and clinical skills training. The goal of these pre-trip activities is for students to gain background knowledge on the region and to become prepared to face the potential challenges and issues unique to working on the US-Mexico border. While in Nogales, AZ students interact with members of the Nogales community including local health center educators and emergency services providers. The students are also exposed to Native American, rural, and migrant issues through their interactions with members of the Tohono O’odham nation, non-profit organization leaders, local and border law enforcements service personnel, and migrants attempting to cross the US-Mexico border. The students also provide service through the coordination of preventative health-screening fairs for older adults in conjunction with a local federally qualified health center. The goal of this study was to assess both the perceived long-term and short-term impact of the one-week service-learning trip in Nogales, Arizona.

## Methods

This study used a mixed qualitative-quantitative methodology to determine the short-term and perceived long-term program impact on student awareness, perceptions and attitudes related to rural, border, immigration, and Native American health. Each student was required to complete weekly journal entries during their preparatory curriculum and daily journal entries throughout their week in Nogales. This study was reviewed and exempted by the Icahn School of Medicine at Mount Sinai Institutional Review Board.

### Short-term

To determine the short-term impact of the Nogales service-learning trip, we conducted a qualitative analysis of journal entries written by participants during their week in Nogales, AZ in 2016. Prior to analysis, all entries were de-identified and stored in NVivo v11.

Entries were analyzed using a grounded theory approach. Two of the investigators independently reviewed a subset of journal entries to identify emerging themes related to the students’ knowledge, attitudes, and perceptions of border and immigration issues and rural and Native American health. The two investigators then reviewed their codebooks along with a third investigator and together reached a consensus of the major themes. This final codebook was then applied in an iterative process to the previously reviewed and the remaining journal entries.

After all of the entries were coded, the investigator’s codebooks were compared using the generation of a κ statistic and the comparison of percent agreement and disagreement. Codes that had greater than a 10.0% disagreement were reviewed and rectified by these same investigators. Subsequently, the total number of sentences, number of sentences codes, and frequency of each code was calculated. All coding and κ statistic calculations were carried out by the NVivo v11 software (QSR International, Melbourne, Australia).

### Long-term

To assess how participants perceived the long-term impact of a domestic service-learning trip we generated a survey based on the common themes identified in the qualitative analysis and sent it to all trip participants from 2009–2016. The survey, consisting of 28 items, was administered via Survey Monkey (Table 1). The first 25 items asked the participants to indicate their level of agree/disagreement on a 5-point Likert scale with statements derived from the learned themes identified in the qualitative portion of the study. The last three items of the survey were open-ended questions asking about the strengths and weaknesses of the trip, and ways to improve the experience. Sixteen out of 25 items on the questionnaire were divided into broad categories of “Impact on Medical Education” and “Impact on Medical Career”. The remainder of the items, including the three open-ended items, was for internal purposes to determine ways to improve this service trip at the Icahn School of Medicine at Mount Sinai.

**Table 1:** Summary of survey questions that were distributed to 84 previous participants of a one-week service learning-trip in Nogales between 2009–2016, 2016.


Please choose to what extent you agree/disagree with the following statements (1–25):	
	Strongly Disagree, Somewhat Disagree, Neither Agree/Disagree, Somewhat Agree, Strongly Agree
1.	My educational experiences on this trip matched my expectations.
2.	I enjoyed the Nogales service-learning trip.
3.	I had positive interactions with the people I encountered in Nogales.
4.	While on the trip, I felt like I was able to draw connections between what I saw/experienced in Nogales and my previous experiences/knowledge.
5.	This trip increased my clinical knowledge base.
6.	This trip enhanced my ability to speak Spanish.
7.	This trip enhanced my ability to educate patients.
8.	This trip enhanced my ability to communicate with patients.
9.	I was exposed to a variety of local opinions on community issues.
10.	The trip enhanced my knowledge of rural health.
11.	The trip enhanced my knowledge of Native American health.
12.	The trip enhanced my knowledge of issues that are unique to border communities.
13.	I learned about the importance of involving the local community in the development/implementation of community programming.
14.	During the trip, I thought about how a person’s motivations for his/her actions should be taken into consideration when assessing the outcomes of his/her actions.
15.	My participation in the Nogales trip had an impact on the Nogales community.
16.	I saw and learned about programs that had an impact on the local community.
17.	I am grateful for being able to participate in the Nogales service-learning trip.
18.	I had experiences on this trip that made me aware of my privilege of access and/or social position as a medical student.
19.	This trip made me more aware of the prevalence of socioeconomic privilege within our society based on race, economics, and/or social position.
20.	This trip enhanced my medical education.
21.	My experience in Nogales will help me/helped me be a better clinician.
22.	My experience on this trip has led me to consider working/work in a rural area.
23.	My experience on this trip has led me to consider working/work in an area with limited healthcare resources.
24.	My experience on this trip has led me to consider pursuing/pursue a career in primary care specialties.
25.	I continue to think about what I experienced and what I learned in Nogales.
What are some strengths of the Nogales Service learning trip?	
What are some weaknesses of the Nogales Service learning trip?	
What are some suggestions for improvement?	


Authors EK and BAC, who had previously participated on the Nogales service-learning trip, were excluded from participation in this study. Data were extracted by EK and BAC.

## Results

### Short-term Impact

All students (n = 7) who participated on the Nogales service-learning trip in 2016 completed a total of 35 journal entries. There were 958 sentences, of which 667 were coded (~70.0% coverage). The κ statistic for EK’s and BAC’s codebooks was 0.7, which according to Landis and Koch is substantial [11]. The percent agreement ranged from 80.0–99.7%, and the percent disagreement ranged from 0.3–20.0%. There was a total of 196 instances where there was a greater than 10% disagreement and all were rectified via discretion of EK and BAC.

The qualitative analysis revealed seven themes, which were grouped in two major categories: explicit and implicit lessons and insights. Explicit insights were defined as tangible lessons or skills gained by the participants. Meanwhile, implicit insights were defined as lessons that students gained through reflection. The themes and categories are summarized in (Table 2).

**Table 2:** Themes and selected statements from 35 journal entries that were completed by medical students during their one-week service learning-trip in Nogales, 2016.

Theme	% of Reflections	Sample journal entries

**Explicit lessons and insights**		

Personal benefit (i.e. improving clinical knowledge, connecting previous experiences with new ones, and increasing awareness about Native American, migrant and rural health issues)	100.0	“Understanding their approach to migrant justice was really interesting because it was very similar to harm reduction principles in medicine.”
Felt that experiences would influence their future medical career	42.8	“I was thinking that I might be able to work in a rural community, which surprised me because I had previously thought that I might only work in rural communities abroad, with a focus on more urban medicine or practicing in a more metropolitan area in my future career.”
Expression of gratitude for educational/clinical/professional exposures or impact on community	37.1	“Unfortunately, in medical school, it’s rare to have an opportunity to discuss and reflect upon our profession so intensely, and I’m immensely thankful we were lucky enough to have this chance, and with such great mentors contributing and facilitating.”

**Implicit lessons and insights**		
Shaped how they view privilege, both personal and observed in members of the community	40.0	“…as physicians with so much social capital and privilege, we have a responsibility to advocate for radical change to best serve the needs of our patients.”
Inspired to continue reflecting on experiences after returning home from trip	34.2	“This makes me think about what it means to meaningfully impact the health of a patient and the teamwork that is necessary to have substantial change.”
Perceived impact on the community	34.2	“I think I was able to convince some people how important it was to cut down on their sugar intake.”
How do motivations impact value of another’s action	22.8	“I believe that someone’s views and opinions do matter, especially when they hold so much economic and social power. It is not enough to say ‘he does good work,’ because to be very honest, he is a wealthy white man who has a lot of influence in a low-income community of color.”

#### Explicit Lessons and Insight

All of the journal entries described how the trip influenced their medical education, whether it was through learning about social determinants of health in Nogales, improving their clinical skills, or drawing connections to concepts they had studied in the past (Table 2). Students reported being introduced to issues unique to life on the border, Native American health, and rural health in 88.6% (n = 31) of entries. Also, in 54.3% (n = 19) of their entries, the students described how their interactions with members of the community provided them with a deeper understanding of how the local community viewed life on US-Mexican border and how various members of the community would come together to improve the overall collective health. In addition to learning about how the social environment influences health in Nogales, AZ, 37.1% (n = 13) of students’ entries revealed how this weeklong service-learning trip enhanced their clinical knowledge, patient communication, and patient education skills. Of the entries, 14.3% (n = 5) also confirmed that it allowed them to improve their Spanish-speaking abilities. In 51.4% (n = 18) of entries, students made connections between what they had learned about or experienced in the past and what they learned about or experienced while in Nogales (data not shown).

While all of the students either directly or indirectly reported that this short-term service-learning trip influenced their future, in 42.8% (n = 15) of journal entries, they explicitly wrote that these experiences may influence their practice of medicine in the future (Table 2). For instance, one of the students, who is interested in surgery, discussed how this trip introduced him to the idea of practicing medicine in a rural community.

Ultimately, many of the students were grateful for their experiences in Nogales, with 37.1% (n = 13) of journal entries including passages about the participants’ gratitude for the trip’s impact on the community, their clinical/professional exposure, or their educational experiences (Table 2). For example, one of the students commented on how she was thankful for having the opportunity to witness a Native American traditional blessing.

#### Implicit Lessons and Insight

In addition to providing the students with tangible knowledge and skills, many of the activities that the participants engaged in made them reflect on how they perceived themselves, their surroundings, and their experiences. During the service portion of the trip, the students coordinated health screenings and volunteered with the Kino Border Initiative in Mexico – an aid center and for recently deported migrants. 31.4% (n = 11) of journal entries contained statements reflecting on how participants felt they impacted the local community (Table 2). Some of the students reported that they felt they had a positive impact, while others felt that they had a negligible impact. For instance, one of the students described his experience speaking with immigrants who had recently crossed the border as “voyeuristic”.

In descriptions of their interactions with members of various community programs, students also reflected upon how they felt these programs influenced the community they serve in 34.2% (n = 12) of entries (Table 2). In 22.8% (n = 8) of entries the students discussed how these experiences made them think about the importance of taking into account an individual’s motivations underlying his/her actions when evaluating his/her impact (Table 2).

Additionally, students commented that their experiences shaped how they viewed privilege, both personal and observed in members of the community, in 40.0% (n = 14) of entries (Table 1). For instance, when reflecting on his experience speaking in one of the local health fairs, one participant realized the unique position of privilege he has as a medical student.

In 34.2% (n = 12) of journal entries, students suggested that they will continue to process their experiences upon their return from the service trip (Table 2). For example, a student began to question our current healthcare system in response to learning about community health workers in Nogales.

### Long-term impact

Out of the total 84 former Nogales participants from 2009–2016, we received 48 responses – a response rate of 57.1%.

#### Impact on Medical Education

Overall, 97.9% (n = 47) of respondents agreed that the Nogales service-learning trip enhanced their medical education. Specifically, respondents agreed that it enhanced their ability to educate patients (n = 41, 85.4%), improved their ability to communicate with patients (n = 39, 81.3%), increased their clinical knowledge base (n = 34, 70.8%), and improved their ability to speak Spanish (n = 25, 52.1%) (Table 3). Similarly, the majority of respondents agreed their participation in this weeklong service-learning trip enhanced their knowledge of issues related to rural (n = 48, 100%), migrant (n = 46, 95.8%) and Native American health (n = 34, 70.8%) (Table 3). Many previous participants also agreed that their experiences provided them with lasting intangible lessons, such as the importance of involving community members in the development of community programs (n = 45, 93.8%), acknowledging an individual’s motivations when assessing his/her actions (n = 32, 66.7%), and becoming more aware of their privilege of access/social position (n = 47, 97.9%) and societal privilege as a whole (n = 45, 93.8%) (Table 3).

**Table 3:** Survey responses received from 48 previous students who participated on the one-week service learning-trip in Nogales, Arizona between 2009–2016.

	Statement	Agree (%)	Disagree (%)

Impact on Medical Education	“…enhanced my medical education.”	47 (97.9)	1 (2.1)
	“…enhanced my ability to educate patients.”	41 (85.4)	7 (14.6)
	“…enhanced my ability to communicate with patients.”	39 (81.3)	9 (18.7)
	“…increased my clinical knowledge.”	34 (70.8)	14 (29.2)
	“…improved my ability to speak Spanish.”	25 (52.1)	23 (47.9)
	“…enhanced my knowledge regarding rural health.”	48 (100.0)	0 (0.0)
	“…enhanced my knowledge regarding migrant health.”	46 (95.8)	2 (4.2)
	“…enhanced my knowledge regarding Native American health.”	34 (70.8)	14 (29.2)
	“…learned about the importance of involving the local community in the development/implementation of community programming.”	45 (93.8)	3 (6.2)
	“…thought about how a person’s motivations for his/her actions should be taken into consideration when assessing the outcomes of his/her actions.”	32 (66.7)	16 (33.3)
	“…made me aware of my privilege of access and/or social position as a medical student.”	47 (97.9)	1 (2.1)
	“…made me more aware of the prevalence of socioeconomic privilege within our society based on race, economics, and/or social position.”	45 (93.8)	3 (6.2)

Impact on Medical Career	“…will help me/helped me be a better clinician.”	44 (91.7)	4 (8.3)
	“…has led me to consider working/work in a rural area.”	28 (58.3)	16 (41.7)
	“…has led me to consider working/work in an area with limited healthcare resources.”	38 (79.2)	10 (20.8)
	“…has led me to consider pursuing/pursue a career in primary care specialties.”	25 (52.1)	13 (47.9)

#### Impact on Medical Career

The majority of respondents also agreed that the weeklong service trip guided them to think more broadly about potential career options and future service opportunities. For instance, respondents agreed that their experience either will help them or has helped them be better clinicians (n = 44, 91.7%), led them to consider working in an area with limited healthcare resources (n = 38, 79.2%), led them to consider working in a rural area (n = 28, 58.3%), or led them consider a career in primary care (n = 25, 52.1%) (Table 3).

## Discussion

Our qualitative analyses revealed that current trip participants reported both short-term and long-term positive impacts on clinical skills and knowledge of rural, Native American, and migrant issues. Additionally, findings from the longitudinal survey administered to all previous participants on the Nogales service-learning trip suggested that this formative one-week experience may have lasting impacts on participant’s medical education and career choices. Our findings are consistent with these previous studies of short-term experiences [10,12] as well as findings from studies assessing international trips longer than one week in duration [3,4,5,7,8,9]. This suggests that domestic short-term service-learning trips provide medical students with experiences that influence their medical education and career, both in the short-term and possibly in the long-term, without the additional logistical obstacles associated with international long-term service-learning trips.

Many of the participants commented on intangible insights such as becoming more aware both of their privilege as a medical student as well as the prevalence of socioeconomic privilege throughout society revealing that the educational impact of this trip went beyond those aims explicitly described. The identification of these concepts through written journal reflections reinforces the idea that in-depth reflection is a cornerstone of impactful service-learning [13,14]. It has been suggested that individuals participating in formative experiences tend to focus on the tangible benefits such as clinical skills or knowledge; however, through in-depth reflection, participants are able gain a better understanding of themselves and their surroundings [10,12].

In addition to the impact on the participants, it is also important to consider the impact of the service-learning trip on the local community. While our study did not directly assess how the local community perceived the impact of the service-trip, many of the entries contained reflections on how the participants perceived their impact on the community. Many of the participants felt that they had a limited impact and felt the need for more follow-up, an idea also expressed by several former participants on the survey as a suggestion for improvement. This sentiment is shared by participants of other service-learning trips in the literature [10]. Several studies have suggested that educational exposure gained by the service-learning participants may be gained at the expense of exploiting vulnerable populations [15,16,17,18]; however, studies directly assessing local community perceptions are limited [19]. These findings in conjunction with our study supports the importance of developing service-learning programs that engage the local community and foster longitudinal collaborative relationships in order to safeguard against exploiting vulnerable populations.

Our study has several limitations. First, the journal entries came from a small cohort (n = 7). However, other studies have reached thematic saturation with similarly small sample sizes [7,10,12]. Furthermore, our findings from our quantitative analysis, which included 48 previous participants, reinforced our findings from the journal entries.

Second, given the unique focus of the Nogales short-term service-learning trip, our findings may be less generalizable to other short-term service-learning trips that are offered at other institutions. However, given the variability among service-learning trips, this limitation is not unique to our study [3,4,5,7,12]. Furthermore, to our knowledge, this is the first study that has evaluated the educational impact of domestic global health experiences; therefore, our study provides valuable insight into the potential benefits of short-term domestic service trips.

Third, the 57.1% (n = 48) response rate to the longitudinal survey may miss additional insights from non-respondents. We attempted to reduce the non-response by routinely following up with previous participants at least three times over the course of one month.

Fourth, given the retrospective and subjective nature of this survey it is possible that recall bias may have influenced the results. The survey also included responses from individuals at different stages in their medical career. Therefore, it was difficult to adequately discern whether this trip had an impact on participants’ career choices or will influence their career choices.

Lastly, similar to previously published studies, our study did not have a control group in its quantitative portion. The presence of a control group would have strengthened and confirmed the suggested association we found between medical career choices and participation on this domestic service learning trip [3,4,9,12].

## Conclusions

This study is unique because it attempts to assess both the short-term and perceived long-term impacts of a short-term service-learning trip in the US. Our study suggests that domestic short-term service-learning experiences can provide a valuable window into global health issues without the logistical issues of having to travel outside the US. Additionally, our findings suggest that there is lasting value in creating challenging short-term educational opportunities for students. It also stresses the value of reflection in service-learning trips to provide participants with opportunities for students to explore and enhance their self-awareness. Future studies assessing the perceptions of the local Nogales community of the weeklong service-learning trip would provide valuable insight into ways to strengthen the partnership. As the number of global health programs continues to grow, it is important to develop additional short-term domestic service-learning trips and continue to expand evaluation efforts to ensure benefit for both participants and the local community.
